# Associated factors for early pregnancy-related anxiety: a multicenter cross-sectional study in Japan

**DOI:** 10.1186/s12884-026-08995-5

**Published:** 2026-03-28

**Authors:** Ritsuko Shirabe, Tsuyoshi Okuhara, Hiroko Okada, Takahiro Kiuchi

**Affiliations:** 1 University Hospital Medical Information Network (UMIN) Center, The University of Tokyo Hospital, Tokyo, 113-8655 Japan; 2 Department of Health Communication, School of Public Health, Graduate School of Medicine, The University of Tokyo, Tokyo, 113-8655 Japan

**Keywords:** Pregnancy-related anxiety, Pregnancy-specific anxiety, Environmental stressors, Anxiety, Self-image, Psychosocial factors

## Abstract

**Background:**

Pregnancy-related anxiety appears to be more strongly associated with adverse outcomes in obstetrics and mental health than commonly assessed general anxiety. Support for anxiety should start in the early stages of pregnancy; however, the associated factors for pregnancy-related anxiety in early pregnancy remain unclear. This study aimed to clarify the associated factors for pregnancy-related anxiety in the early stages of pregnancy.

**Methods:**

We conducted a cross-sectional study among 183 pregnant Japanese women who were ≥ 18 years, pregnant with one baby, and before 15 weeks of gestation from four facilities in Japan. Pregnancy-related anxiety was assessed using the Japanese version of the Pregnancy-Related Anxiety Questionnaire-Revised-2. Multiple linear regression analysis was performed to identify factors associated with total and subscale of pregnancy-related anxiety, including obstetric, individual, and environmental factors.

**Results:**

The model that included all factors accounted for 24% of the variance in total pregnancy-related anxiety score. Significant associated factors included previous deliveries (β = -0.27), general anxiety (β = 0.26), a history of smoking (β = 0.19), and pre-pregnancy body mass index (β = 0.17). Lifetime history of abuse and/or domestic violence (β = 0.20) and current daily hassles (β = 0.20) were also independently associated with higher anxiety levels. Subscale analysis revealed a distinct pattern: while traditional obstetric and individual factors were associated with fear of childbirth and worries about bearing a handicapped child, environmental stressors were specifically associated with concern about own appearance.

**Conclusions:**

This study demonstrated that pregnancy-related anxiety is uniquely shaped by the additive effects of lifetime trauma and current environmental stress. While traditional clinical backgrounds help identify fears regarding childbirth and infant health, they are insufficient to capture self-image concerns. In addition to identifying a history of abuse or high daily stress, we recommend direct measurements of pregnancy-related anxiety to provide tailored counseling that addresses these often-overlooked psychological vulnerabilities in early pregnancy.

**Supplementary Information:**

The online version contains supplementary material available at 10.1186/s12884-026-08995-5.

## Introduction

Perinatal mood and anxiety disorders are the most common complications of pregnancy and are associated with adverse outcomes such as perinatal suicidality [1]. Although risk factors and interventions for perinatal mood and anxiety disorders have been established, the deterioration of maternal mental health is still an ongoing issue worldwide, with an increase in suicidal ideation in some countries [2]. Current screening and interventions are limited in their ability to protect all women during the perinatal period; therefore, different and distinct preventive approaches are needed.

Among several anxiety types, pregnancy-related anxiety is a content-specific form of anxiety that focuses on concerns about pregnancy, childbirth and parenting [3]. Although pregnancy-related anxiety overlaps with general anxiety, previous studies have demonstrated only moderate correlations with state and trait anxiety, suggesting that it represents a related but distinct construct [4, 5]. Pregnancy-related anxiety is more strongly associated than general anxiety with postpartum affective disturbances, such as parenting stress, anxiety disorders, and postpartum depression [6–8], as well as adverse obstetric outcomes (e.g., preterm birth) [9]. Previous studies have reported heterogeneous trajectories of pregnancy-related anxiety across gestation, including downward [10], U-shaped [11, 12], and stable patterns [13]; however, elevated anxiety levels during early pregnancy have been observed across these studies. Early stage of pregnancy is also clinically characterized by heightened uncertainty, perceived risk of miscarriage, and pronounced physical symptoms such as strong hyperemesis, which may amplify psychological distress [14]. Therefore, early pregnancy was considered a critical period for investigation.

A systematic review revealed a significant association between antenatal general anxiety and several related factors, including obstetric, individual, and environmental factors [15]. With respect to pregnancy-related anxiety, previous studies have reported several associated factors, such as nulliparity, medical complications, unplanned pregnancy, younger age, lower education, being unmarried, stressful life events, and perceived stress [12, 16–19]. However, these studies have not shown consistent results. Although the levels of pregnancy-related anxiety may change throughout pregnancy [11, 12], most previous studies were conducted with a cross-sectional design and included women at different stages of gestation. It is essential to identify pregnancy-related anxiety and start providing support from the early stages of pregnancy. Although direct screening is important, systematic screening for pregnancy-related anxiety is not always implemented in routine clinical practice, and healthcare providers often rely on background characteristics to assess vulnerability. However, the background factors associated with pregnancy-related anxiety in early pregnancy remain unclear.

This study aimed to identify the background factors associated with high levels of pregnancy-related anxiety in the first trimester of pregnancy using cross-sectional data from pregnant Japanese women. We hypothesized that identifying these factors would provide insights for clinical assessment strategies and support the development of appropriate early interventions for pregnancy-related anxiety.

## Methods

### Settings and participants

We conducted this cross-sectional study as a part of a longitudinal study that followed women’s emotional responses from pregnancy to the postpartum period. The present study was performed at two facilities in Tokyo (one regional perinatal medical center and one city hospital) and two clinics in Kanagawa. Outpatients who were Japanese, aged ≥ 18 years, pregnant with one baby, in early pregnancy (< 15 weeks), had no problems reading Japanese and were planning to deliver at these facilities were eligible for this study. To minimize selection bias, recruitment was conducted through consecutive sampling at multiple facilities with differing characteristics without excluding high-risk groups. Participants were recruited during regular checkups by the first author or research collaborators (midwives) at each facility between April 2022 and May 2023. Written informed consent to participate and publish the study was obtained from all participants after providing both oral and written explanations of the study. Participants were assured that participation was voluntary and would not affect their medical care. Questionnaire collection were conducted either directly by midwives or administrative staff or by mail to the research team, and the attending obstetricians were not involved in the process. We sent participants a 1,000-yen (roughly equivalent to USD 8) gift certificate in return for the last (postpartum) questionnaire in the longitudinal study. To ensure anonymity, completed questionnaires were placed in sealed envelopes by the participants before collection. The cover sheet containing participants’ names was removed and destroyed after assigning a research identification number. The anonymized data were stored securely and were accessible only to the research team. To encourage candid disclosure of sensitive information, individual responses were kept confidential and were not shared with the clinical staff. However, the study protocol ensured participant safety; the informed consent process specified that researchers would provide information on specialized counseling services or facilitate referrals to professional support if a participant’s responses indicated a need for urgent intervention. This study was approved by the Institutional Review Board of the University of Tokyo (approval code: 2020154NI).

### Sample size

Using G*Power 3.1 [20], the required total sample size for the study was calculated as 160. This calculation was based on a two-tailed test with a power (1-β) of 0.8 and α = 0.05, with an effect size set at 0.15 and 21 independent variables described below. Considering the missing values, this study determined a sample size of 200.

### Measures

We used the questionnaire which was developed for this study (Additional file 1).

#### Dependent variable

We measured pregnancy-related anxiety as the main outcome by using the Japanese version of the Pregnancy-Related Anxiety Questionnaire-Revised-2 (PRAQ-R2), which is a reliable and validated scale for assessing pregnancy-related anxiety regardless of parity [21]. This is a 10-item self-report measure with three factors similar to those in the original study [11]: fear of giving birth (FOGB), worries about bearing a handicapped child (WBHC), and concern about one’s own appearance (COA). The items are rated from 1 (absolutely not relevant) to 5 (very relevant), and a higher total score indicates more severe anxiety (total score range: 10–50). In a previous longitudinal validation study, this scale demonstrated good predictive validity for screen-positive postnatal depressive symptoms, with an Area Under the Curve of 0.74 in early pregnancy [8]. Women scoring above a threshold of 34 in early pregnancy were found to have a 3.4-fold increased relative risk (RR = 3.4, 95% CI: 1.4–8.2) of screen-positive postnatal depressive states [8]. In this study, the total PRAQ-R2 score was analyzed as a continuous variable with the omega total value 0.91 for the overall PRAQ-R2, which supported very good internal consistency. The omega totals for the three factors were as follows: FOGB, 0.80; WBHC, 0.95; and COA, 0.77.

#### Independent variables

We set the variables with reference to systematic reviews of risk factors for antenatal anxiety and postpartum depression [15, 22]. For obstetric factors, data on the number of previous deliveries and abortions, current or past complications, and means of conception were obtained from medical records. The participants reported their pregnancy expectations in the questionnaire with three choices: expected, unplanned, and unwanted.

The psychological, psychiatric, sociodemographic, and economic characteristics of the participants were evaluated as individual factors. General anxiety was measured via the validated Japanese version of the Hospital Anxiety and Depression Scale-Anxiety (HADS-A) [23], which was developed by referring to the original scale that can assess anxiety symptoms [24]. This scale consists of seven items rated on a four-point Likert scale ranging from one to four. The omega total for HADS-A in this study was 0.81. We assessed prenatal depressive symptoms via the Japanese version of the Edinburgh Postnatal Depression Scale (EPDS) [25], developed by referring to the original scale, which can assess perinatal depressive states [26]. This is a validated and reliable 10-item self-report measure, and each item is rated on a four-point Likert scale ranging from zero to three. We utilized the total EPDS score as a continuous variable to maximize statistical power and preserve the dose-response relationship, rather than using a binary cutoff. The omega total for the EPDS in this study was 0.88. The participants also answered questions on academic background, job, and socioeconomic status (annual household income and economic comfort). We obtained data on age, history of smoking, alcohol use during pregnancy, and height and weight before pregnancy from medical records.

Based on the previous reviews [15, 22], we evaluated two types of environmental stressors: current stress (e.g., during the present pregnancy) and lifetime stress (e.g., history of abuse/ domestic violence (DV)). This approach allowed for a comprehensive assessment of the participant’s psychological environment across different temporal stages. We assessed current perceived stress via the 30-item Daily Hassles Scale developed in Japan [27] with reference to the original scale [28]. Higher scores indicate greater levels of daily hassles. The omega total for this scale in this study was 0.90. A history of abuse and/or DV were assessed as an adverse event in life using a single binary question: “Have you ever experienced abuse or domestic violence in your lifetime?” We also evaluated social support as environmental factors via the Japanese version of the Social Support Questionnaire (J-SSQ) [29] with reference to the original scale [30]. The J-SSQ comprises 12 items and has two factors: the number of persons (NP) and the satisfaction rating (SR). NP is the sum of the perceived numbers of available others in six different situations. The SR is the sum of the degree of satisfaction in these six situations, ranging from 1 (very dissatisfied) to 6 (very satisfied). The overall omega total and two factors (NP and SR) of the J-SSQ were 0.90, 0.92, and 0.98, respectively.

### Statistical analysis

We performed multivariate regression analyses with pregnancy-related anxiety (overall PRAQ-R2 score) as the dependent variable and background characteristics as the independent variables for forced entry. First, we performed a simple linear regression analysis between each independent variable and pregnancy-related anxiety, followed by an examination of the associations between each independent variable. After confirming multicollinearity by calculating the variance inflation factor (VIF), we performed a hierarchical multiple linear regression analysis. In Model 1, we included only the independent variables of obstetric- and pregnancy-related factors: previous delivery (number), previous abortion (yes/no), current/past complications (yes/no), infertility treatment (yes/no), and unintended pregnancy (yes/no). Pregnancy intention was re-categorized into two groups: ‘intended’ and ‘unintended’ (combining ‘unplanned’ and ‘unwanted’ pregnancies). This consolidation addressed the sparseness of the ‘unwanted’ category (*N* = 2) and improved the robustness of the regression estimates. In Model 2, we added the individual factors: general anxiety (continuous), prenatal depressive symptoms (continuous), history of mental illness (yes/no), current use of alcohol (yes/no), smoking before or during pregnancy (yes/no), age (continuous), years of education (junior high school or high school, junior college or vocational/technical school, bachelor’s degree, postgraduate degree), working full-time (yes/no), economic comfort (continuous), and body mass index (BMI) before pregnancy (continuous). We chose economic comfort as the economic variable because annual income exhibited a ceiling effect (Table 1). Regarding employment, we determined whether individuals were working full-time rather than simply whether they were employed as variables because a simple regression analysis indicated that working full-time rather than unemployed tended to result in greater pregnancy-related anxiety in this sample (b = 1.6, *p* = 0.15), which was in line with a previous study that reported an association between job stress and pregnancy-related anxiety [18]. In Model 3, we added the following environmental factors: daily hassles (continuous), history of abuse and/or DV (yes/no), and social support (continuous). Independent variables that showed a right-skewed distribution (general anxiety, daily hassles, and social support (NP)) were log-transformed to meet the assumption of normality for linear regression analysis. We confirmed that the residuals of the assumed model were approximately normally distributed via a Q‒Q plot. Potential influential observations were evaluated using Cook’s distance, with a cut-off value of 1.0 to ensure that no single case unduly influenced the model results. We assessed the applicability of each model via the adjusted coefficient of determination (adjusted R^2^) and examined the validity of the added variables according to the degree of change in the coefficient of determination (ΔR^2^).

**Table 1:** Characteristics of the participants (*N* = 183)

Variables	*N* = 183		%
Age (years), median (IQR^a^)	33 (31–36)		
Previous delivery, mean (SD^b^)	0.73 (0.68)		
Previous abortion			
Yes	47		26
Current/past complications			
Yes	64		35
Means of conception			
Natural	161		88
Artificial insemination	4		2
In vitro fertilization	17		9
Syringe (donor sperm)	1		0.5
Pregnancy expectation			
Expected	161		88
Unplanned	20		11
Unwanted	2		1
General anxiety, median (IQR^a^)	4.0 (2–7)		
Prenatal depressive symptoms, median (IQR^a^)	3.0 (1–6)		
History of mental illness			
Yes	13		7
Current use of alcohol			
Yes	10		6
Smoking status			
Current	1		0.5
Quit after pregnancy	15		8
Education			
Junior high school or high school	15		8
Junior college or vocational/technical school	40		22
Bachelor’s degree	116		63
Postgraduate degree	12		7
Full-time work			
Yes	140		77
Economic comfort			
Very little	5		3
Not much	45		25
Some	128		70
Considerable	5		3
Annual household income (JPY^c^)			
< 2.0 million	2		1
2.0–4.0 million	13		7
4.0–5.9 million	24		13
6.0–7.9 million	31		17
8.0–10 million	43		24
> 10 million	70		38
Body mass index before pregnancy (kg/m^2^), median (IQR^a^)	21 (19–22)		
Daily hassles, median (IQR^a^)	0 (0–2)		
History of abuse and/or domestic violence			
Yes	8		4
Social support			
Number of persons, median (IQR^a^)	22 (17–30)		
Satisfaction rating, median (IQR^a^)	30 (29–36)		
Pregnancy-related anxiety (the PRAQ-R2 score), mean (SD^b^)			
Total	32.4 (6.8)		
Fear of giving birth	9.8 (2.7)		
Worries about bearing a handicapped child	14.3 (3.9)		
Concern about one’s own appearance	8.4 (2.9)		

^a^Interquartile range

^b^Standard deviation

^c^Roughly equivalent to USD 0.007

Furthermore, we analyzed the associations between the independent variables and each subscale of the PRAQ-R2 to evaluate the different associations among subscales.

All the statistical analyses were performed using R for Windows (version 4.3.1; R Foundation for Statistical Computing, Vienna, Austria). The significance threshold was set at *p* < 0.05. If the number of missing data points was sufficiently small, a complete case analysis was performed.

## Results

### Participant characteristics

Among the 227 women recruited for this study, 201 who answered the questionnaires at a median pregnancy of nine weeks (interquartile range (IQR): 8.5–10) were included. Twenty-six women were excluded from the analysis: four women were transferred to different hospitals, two were hospitalized, one withdrew consent owing to strong anxiety, three provided responses at an inappropriate stage, and 16 did not return the questionnaire. To ensure the stability of the statistical inference, we limited the primary analysis to partnered/married participants without missing data (*N* = 183). The number of single/unpartnered individuals was extremely small (*N* = 2), and their inclusion would have resulted in unstable estimates and potential bias due to insufficient power for that subgroup.

Table 1 presents the participants’ characteristics. Common current/past complications included asthma (*N* = 20), thyroid disease (*N* = 8), previous cesarean section (*N* = 7), leiomyoma (*N* = 5), and gestational diabetes (*N* = 5) (there were overlaps between some of these conditions). Past mental illnesses included maladjustment and/or panic disorder (*N* = 9), depression (*N* = 6) and a sleep disorder (*N* = 1) (there was an overlap between these conditions). All variables had < 5% missing values (maximum of 10 for social support (SR)), supporting the adoption of a complete case analysis. Comparison between included and excluded participants showed no significant differences in the primary outcome (pregnancy-related anxiety), although a minor difference in educational level was noted, suggesting limited selection bias. The Shapiro–Wilk normality test indicated that the total score of the PRAQ-R2 exhibited a normal distribution (*p* = 0.63), whereas the scores of each subscale exhibited a nonnormal distribution.

### Associations between overall pregnancy-related anxiety and background factors

The maximum VIF across all independent variables was 2.2, indicating no multicollinearity. Table 2 shows the results of the hierarchical linear regression analysis, which tested the associations between background characteristics and overall pregnancy-related anxiety. Model 1, which included only obstetric and pregnancy-related variables, explained only 8.4% of the variance in pregnancy-related anxiety. Model 2, in which individual factors were added as independent variables, explained an additional 17% of the variance in pregnancy-related anxiety. In Model 3, we added environmental factors as independent variables. Model 3 explained an additional 5.6% of the variance in pregnancy-related anxiety. In Model 3, previous delivery was associated with decreases in overall pregnancy-related anxiety scores, respectively (standardized β = -0.27). In addition to a history of smoking (β = 0.19), BMI before pregnancy (β = 0.17), and general anxiety (β = 0.26), environmental stressors were positively associated with overall pregnancy-related anxiety; daily hassles (β = 0.20) and experiences of abuse and/or DV (β = 0.20). The Q‒Q plots of the analysis indicated that the residuals of the assumed model were approximately normally distributed. The maximum value of Cook’s distance was 0.12, ensuring the stability and robustness of our findings.

**Table 2:** Factors associated with pregnancy-related anxiety (PRAQ-R2 total score): Hierarchical linear regression analysis (*N*=183)

Variable	Model 1		Model 2		Model 3	
	b (95%CI^a^)	*p-value*	b (95%CI^a^)	*p-value*	b (95%CI^a^)	*p-value*
Model 1 (obstetric factors)						
Previous delivery	-3.4 (-4.9, -1.9)	**<0.001**	-2.8 (-4.3, -1.2)	**<0.001**	-2.7 (-4.2, -1.2)	**<0.001**
Previous abortion	-0.4 (-2.7, 1.8)	0.69	-0.43 (-2.6, -1.8)	0.70	-0.58 (-2.7, 1.6)	0.60
Current/past complication	0.94 (-1.1, 3.0)	0.36	0.63 (-1.4, 2.7)	0.55	0.57 (-1.5, 2.6)	0.59
Infertility treatment	1.4 (-1.5, 4.4)	0.34	0.50 (-2.4, -3.5)	0.74	0.49 (-2.4, 3.4)	0.74
Unintended pregnancy	2.3 (-0.82, 5.4)	0.15	1.1 (-2.1, 4.3)	0.49	0.85 (-2.3, 4.0)	0.59
Model 2 (Model 1 + individual factors)						
General anxiety^c^			3.4 (1.3, 5.5)	**0.0019**	3.2 (1.1, 5.3)	**0.0033**
Perinatal depressive symptoms			-0.12 (-0.44, 0.20)	0.46	-0.28 (-0.61, 0.061)	0.11
History of mental illness			0.62 (-3.2, 4.5)	0.75	-0.47 (-4.4, 3.4)	0.81
Current use of alcohol			-0.53 (-4.5, 3.4)	0.79	0.088 (-3.9, 4.0)	0.96
History of smoking			4.8 (1.4, 8.3)	**0.0061**	4.5 (1.1, 7.9)	**0.0089**
Age			-0.12 (-0.38, 0.14)	0.38	-0.033 (-0.30, 0.23)	0.80
Education (ref. bachelor’s degree)						
Junior high school or high school			0.42 (-3.1, 4.0)	0.82	-0.27 (-3.9, 3.3)	0.88
Junior college or vocational/technical school			-2.6 (-4.9, -0.31)	**0.026**	-3.0 (-5.2, -0.69)	**0.011**
Postgraduate degree			0.21 (-3.6, 4.0)	0.91	1.5 (-2.4, 5.3)	0.45
Fulltime work			2.5 (0.19, 4.8)	**0.034**	2.2 (-0.091, 4.5)	0.060
Economic discomfort			0.0081 (-1.7, 1.7)	0.99	0.019 (-1.7, 1.7)	0.98
Body mass index before pregnancy			0.50 (0.14, 0.85)	**0.0062**	0.44 (0.088, 0.78)	**0.014**
Model 3 (Model 2 + environmental factors)						
Daily hassles^c^					1.9 (0.36, 3.5)	**0.016**
Abuse/domestic violence					6.5 (1.8, 11.3)	**0.0071**
Social support (number of persons)^c^					2.1 (-0.16, 4.4)	0.068
Social support (satisfaction rating)					-0.055 (-0.20, 0.087)	0.45
F-statistic (*p *value)	(5, 177) = 4.4 (*p*<0.001)		(17, 165) = 3.7 (*p*<0.001)		(21, 161) = 3.8 (*p*<0.001)	
Adjusted R^2^	0.084		0.20		0.24	
ΔR^b^ (*p*value)			0.17 (***p<0.001***)		0.056 (***p=0.011***)	

^a^Confidence interval

^b^Body mass index

^c^Log-transformed (i.e., b represents the change in the PRAQ-R2 score associated with a 1-unit increase in the natural log-transformed variable)

Significant *p*-values (*p* < 0.05) are shown in bold

### Associations between each subscale of pregnancy-related anxiety and background factors

Supplemental material B presents the results of the multiple linear regression analysis that tested the associations between background characteristics and each subscale. Lower delivery experience, general anxiety, and a history of smoking were associated with higher FOGB and WBHC counts but not with COA. In addition to BMI before pregnancy, daily hassles and a history of abuse and/or DV were positively associated with COA. The Q‒Q plots of each analysis indicated that the residuals of the assumed model were approximately normally distributed. The maximum value of Cook’s distance in these analyses was 0.15.

## Discussion

We examined the associations between pregnancy-related anxiety in the first trimester and background characteristics in pregnant Japanese women. The final model accounted for 24% of the variance in pregnancy-related anxiety. In addition to previous delivery experience, general anxiety, a history of smoking, and pre-pregnancy maternal BMI, environmental factors including lifetime history of abuse/DV and current daily hassles were independently associated with pregnancy-related anxiety scores in the early stages of pregnancy. Notably, the impacts of lifetime history of abuse/DV (β = 0.20) and current daily hassles (β = 0.20) were comparable in magnitude to that of well-established contributing factors such as parity (β = -0.27) and general anxiety (β = 0.26).

Consistent with previous findings [12, 17–19, 31], fewer childbirth events and a history of smoking were associated with higher pregnancy-related anxiety scores. Additionally, our results showed that both lifetime abuse/DV and current daily hassles were significantly associated with pregnancy-related anxiety. Notably, there was no significant difference in daily hassle scores between the participants with and without a history of abuse/DV (*p* = 0.48). This lack of association suggests that lifetime stressors and current stressors function as independent associated factors of pregnancy-related anxiety. Lifetime abuse may contribute to psychological vulnerability or heightened baseline of anxiety, which is distinct from the situational stress caused by current environmental demands. According to the diathesis-stress model, prior adverse experiences such as abuse or DV may establish a psychological vulnerability—or diathesis—that predisposes individuals to heightened anxiety, while current daily hassles serve as proximal stressors that trigger or amplify this underlying vulnerability [32]. Rather than historical trauma merely increasing the perception of daily stress (mediation), these two pathways appear to operate independently, each contributing a distinct burden to pregnancy-related anxiety.

As previous studies have suggested [17, 19], each factor has a different association with different types of pregnancy-related anxiety. Traditional clinical and obstetric factors—such as parity and smoking history—were predominantly associated with fear of childbirth and infant health. In contrast, these factors showed no significant association with anxiety about body image. Instead, appearance-related anxiety was specifically linked to environmental stressors, namely lifetime abuse/DV and daily hassles. Fears regarding the delivery process or the baby’s health may be more influenced by clinical or obstetric factors than by long-term psychological stress history. In contrast, lifetime adversity, such as abuse or DV, is well-documented to undermine self-esteem and body image [33–35]. Our results suggest that such past experiences, combined with current daily stressors, may specifically sensitize women to the rapid physical changes of pregnancy as a loss of control over one’s body, leading to heightened appearance-related anxiety. While the explained variance for this subscale was low (adjusted R^2^ = 0.097), this highlights that self-image concerns in early pregnancy are uniquely driven by psychological and historical adversity rather than conventional obstetric profiles.

In this study, the final model accounted for only 24% of the variance in pregnancy-related anxiety, indicating that the variables measured in this study could not explain a large proportion of pregnancy-related anxiety. The adjusted R^2^ was also modest in previous studies, in which the models accounted for 18% and 41% of the variance in pregnancy-related anxiety [16, 17]. Because pregnancy-related anxiety could not be sufficiently explained by background characteristics alone, identifying affected women based solely on background demographic and clinical characteristics may be difficult. Particularly, concerns about one ‘s own appearance can be easily overlooked in existing risk screening. Pregnancy-related anxiety is associated with poor postpartum maternal mental health [6–8] and adverse obstetric outcomes such as preterm birth [9]. Therefore, gathering a history of abuse/DV, adopting an empathetic approach to everyday minor stressors, and direct assessment using validated pregnancy-related anxiety scales is likely necessary in clinical practice. Such proactive screening could facilitate the early identification of women whose anxiety is rooted in past trauma or current living conditions, potentially allowing for timely referrals to psychological counseling or relevant community-based support services.

Although general anxiety is positively associated with pregnancy-related anxiety, its contribution is limited in our model. Additionally, prenatal depressive symptoms were not associated with pregnancy-related anxiety, which is consistent with the results of a previous study [17]. These findings further support the distinction of pregnancy-related anxiety and general anxiety or prenatal depressive symptoms [4]. Even in countries where screening for antenatal anxiety is recommended, scales for general anxiety are usually used [36]. However, given the distinct nature of pregnancy-related anxiety demonstrated in this and previous studies, the use of pregnancy-specific anxiety scales, either alone or in combination with general measures, may be more appropriate.

This study had several limitations. First, the participants in the previous validation study of the Japanese version of the PRAQ-R2 [21] were included as participants in this study because of recruitment difficulties during the COVID-19 pandemic. The scale developed for the validation study was successful and did not require revision. Therefore, the impact of conducting the present study with the participants of the validation study on the interpretation of the results was limited. Second, although this study was conducted at multiple facilities, there may have been selection bias in terms of socioeconomic background, possibly because the study was conducted in an urban area. Further research including more representative samples (e.g., women from lower socioeconomic backgrounds) is needed to improve the generalizability of the results. However, it is noteworthy that even within this relatively socioeconomically stable sample with limited variability, subjective daily stressors and a history of abuse/DV emerged as potent predictors of pregnancy-related anxiety. This suggests that these environmental factors may override the protective effects of a high-resource environment, underscoring the necessity of universal screening for these risks across all populations, regardless of their socioeconomic status. Third, comparison between included and excluded participants showed a minor difference in educational level. To account for the potential influence of educational background, we included it as a covariate in our final regression model. Fourth, although the previous validation study showed good reliability [21], this study was conducted in the early stages of pregnancy when pregnant women are prone to physical changes, which may affect the intraindividual variation in the pregnancy-related anxiety score. Fifth, owing to its cross-sectional design, we cannot establish causal relationships between the identified factors and pregnancy-related anxiety. For instance, while we found an association between current daily hassles and high anxiety, it is possible that individuals with pre-existing high anxiety might perceive or report their environmental stressors more negatively (reverse causation). Similarly, other psychosocial variables could be influenced by the participant’s current psychological state at the time of the survey. Future longitudinal studies are needed to clarify the directional pathways and confirm whether these associated factors truly precede the onset of pregnancy-related anxiety. Sixth, as the study utilized a single binary item focusing on lifetime experiences, it could not distinguish between the timing (e.g., childhood vs. current pregnancy) or the severity of the abuse/DV. This may result in a heterogeneous group within the ‘yes’ category. Furthermore, due to the sensitive nature of abuse and DV, the potential for underreporting cannot be entirely ruled out. However, to minimize this bias and encourage candid disclosure, we strictly maintained participant anonymity throughout the study, ensuring that individual responses were not shared with clinical staff. This confidential research environment may have facilitated more honest reporting of lifetime stressors compared to routine clinical interviews. Future research should utilize validated instruments that capture the timing, frequency, and nature of these experiences to better understand their specific impact on perinatal mental health. Despite these limitations, this study is the first to examine the associations between background factors and pregnancy-related anxiety, which is limited to the early stages of pregnancy.

## Conclusions

This study identified that obstetric characteristics, individual traits, and environmental stressors combine to be associated with pregnancy-related anxiety in the first trimester. Our findings uniquely highlight that while obstetric factors primarily relate to fears about childbirth and the infant’s health, lifetime trauma and current daily stressors were distinctively associated with appearance-related anxiety. This suggests that early pregnancy anxiety is a complex construct where personal history and environmental demands intersect, particularly affecting a woman’s self-concept. Relying solely on conventional clinical backgrounds to identify at-risk women is insufficient; direct screening using pregnancy-specific anxiety scales and sensitive assessment of psychosocial stressors are vital for providing early, tailored support to improve maternal mental health.

## Supplementary Information

Supplementary Material 1.

Supplementary Material 2.

## Data Availability

The datasets analyzed during the current study are available from the corresponding author upon reasonable request. The data are not publicly available because the participants provided their consent after being informed that their personal data would not be disclosed to outside parties and that only the results of the study would be published in academic journals.
