# Supplementary material for: Associated factors for early pregnancy-related anxiety: a multicenter cross-sectional study in Japan
Source: BMC Pregnancy Childbirth. 2026 Mar 28;26:500. doi: 10.1186/s12884-026-08995-5 (PMC13151401; doi:10.1186/s12884-026-08995-5)
Supplement: Supplementary file 1 — Supplementary Material 1. [file 12884_2026_8995_MOESM1_ESM.docx]

* Since the developers of the scales did not give us permission to present them in this paper, we have included only the names of the scales in the questionnaire.

1. The Japanese Version of Pregnancy-Related Anxiety Questionnaire-Revised-2*

2. Please circle the answer that best applies to your situation.

|  | Absolutely not relevant | Hardly ever relevant | Reasonably relevant | Very relevant |
| --- | --- | --- | --- | --- |
| 1. I'm concerned about whether I can take care of a baby. | 1 | 2 | 3 | 4 |
| 2. I don't know if the midwife or doctor will be genuinely caring toward me. | 1 | 2 | 3 | 4 |
| 3. I don't know if the midwives and doctors are kind and will be helpful to me. | 1 | 2 | 3 | 4 |
| 4. I don't know if I can ask midwives or doctors anything. | 1 | 2 | 3 | 4 |

1. The Japanese version of the Hospital Anxiety and Depression Scale—Anxiety*
2. The Japanese version of the Edinburgh Postnatal Depression Scale*
3. The Japanese version of the Prenatal Self-Evaluation Questionnaire*
4. The 30-item Daily Hassles Scale*
5. The Japanese version of the Social Support Questionnaire*

Finally, we would like to ask you about yourself.

Please circle the number that applies.

8. Please tell me your occupation.

(1) Company employee (full-time employee) (2) Company employee (contract employee)　(3) Civil servant　(4) Self-employed/freelancer　(5) Company executives and managers (6) Part-time work　(7) Student　(8) Housewife　(9) Unemployed

9. For those planning to work while raising children: Please circle the option that best describes your work situation since your baby was born.

Job control (subscale from The Brief Job Stress Questionnaire)*

10. Please tell us the last educational institution you graduated from.

(1) Junior high school or high school　（2）National College of Technology or Vocational College or Junior College (3) University (4) Six-year university and graduate school

11. Please tell us your household income.

（1）Less than 2 million yen　（2）2 million to 4 million yen

（3）4 million to 6 million yen　（4）6 million to 8 million yen

（5）8 million to 10 million yen　（6）More than 10 million yen

12. How financially comfortable is your household?

(1) Quite comfortable (2) To some degree　(3) Not very comfortable　(4) With almost no financial leeway

13. Did you expect this pregnancy?

(1) Wanted　(2) Unintended (3) Unwanted

14. Have you ever experienced abuse or domestic violence since you were born?

(1) yes　(2) no
