# Supplementary material for: Associated factors for early pregnancy-related anxiety: a multicenter cross-sectional study in Japan
Source: BMC Pregnancy Childbirth. 2026 Mar 28;26:500. doi: 10.1186/s12884-026-08995-5 (PMC13151401; doi:10.1186/s12884-026-08995-5)
Supplement: Supplementary file 2 — Supplementary Material 2. [file 12884_2026_8995_MOESM2_ESM.docx]

**Supplemental material B.** Factors associated with each subscale of PRAQ-R2: Multiple linear regression analysis (N=183).

| Variable | FOGB^1^ | | WBHC^2^ | | | | COA^3^ | | | |
| --- | --- | --- | --- | --- | --- | --- | --- | --- | --- | --- |
|  | b (95%CI^4^) | *p-value* | b (95%CI^4^) | | | *p-value* | b (95%CI^4^) | | | *p-value* |
| Previous delivery | -1.6 (-2.2, -1.0) | **<0.001** | -1.3 (-2.3, -0.42) | | | **0.0047** | 0.24 (-0.48, 0.95) | | | 0.52 |
| Previous abortion | 0.53 (-0.29, 1.3) | 0.20 | -0.56 (-1.9, 0.76) | | | 0.40 | -0.55 (-1.6, 0.48) | | | 0.29 |
| Current/past complication | 0.80 (0.025, 1.6) | **0.043** | -0.026 (-1.28, 1.2) | | | 0.97 | -0.21 (-1.2, 0.77) | | | 0.67 |
| Infertility treatment | -0.91 (-2.0, 0.18) | 0.10 | 0.98 (-0.78, 2.8) | | | 0.27 | 0.42 (-0.96, 1.8) | | | 0.55 |
| Unintended pregnancy | 0.52 (-0.67, 1.7) | 0.39 | 0.43 (-1.5, 2.4) | | | 0.66 | -0.096 (-1.6, 1.4) | | | 0.90 |
| General anxiety^6^ | 1.5 (0.66, 2.2) | **<0.001** | 1.8 (0.48, 3.1) | | | **0.0074** | -0.041 (-1.0, 0.96) | | | 0.94 |
| Perinatal depressive symptom | -0.068 (-0.20, 0.059) | 0.29 | -0.085 (-0.29, 0.12) | | | 0.42 | -0.12 (-0.28, 0.036) | | | 0.13 |
| History of mental illness | -0.23 (-1.7, 1.2) | 0.76 | -0.55 (-2.9, 1.8) | | | 0.65 | 0.31 (-1.5, 2.1) | | | 0.74 |
| Current use of alcohol | 1.0 (-0.45, 2.5) | 0.17 | -0.84 (-3.2, 1.6) | | | 0.49 | -0.11 (-2.0, 1.8) | | | 0.91 |
| History of smoking | 1.4 (0.15, 2.7) | **0.029** | 2.1 (0.074, 4.2) | | | **0.042** | 0.96 (-0.63, 2.6) | | | 0.24 |
| Age | -0.013 (-0.11, 0.088) | 0.80 | -0.00050 (-0.16, 0.16) | | | 0.995 | -0.020 (-0.15, 0.11) | | | 0.76 |
| Education (ref. bachelor’s degree) | | | | | | | | | | |
| Junior high school or high school | -0.73 (-2.1, 0.63) | 0.29 | 0.65 (-1.5, 2.8) | | | 0.56 | -0.19 (-1.9, 1.5) | | | 0.83 |
| Junior college or vocational/technical school | -0.76 (-1.6, 0.085) | 0.077 | -1.1 (-2.4, 0.31) | | | 0.13 | -1.2 (-2.2, -0.047) | | | **0.041** |
| Postgraduate degree | -0.34 (-1.8, 1.1) | 0.65 | 1.6 (-0.76, 3.9) | | | 0.18 | 0.24 (-1.6, 2.1) | | | 0.79 |
| Fulltime work | 0.66 (-0.21, 1.5) | 0.14 | 1.2 (-0.21, 2.6) | | | 0.094 | 0.35 (-0.74, 1.4) | | | 0.52 |
| Economic discomfort | 0.49 (-0.14, 1.1) | 0.12 | -0.18 (-1.2, 0.84) | | | 0.73 | -0.30 (-1.1, 0.49) | | | 0.46 |
| BMI^5^ before pregnancy | 0.093 (-0.038, 0.22) | 0.16 | 0.13 (-0.086, 0.34) | | | 0.24 | 0.22 (0.051, 0.38) | | | **0.011** |
| Daily hassles^6^ | 0.26 (-0.33, 0.84) | 0.39 | 0.37 (-0.58, 1.3) | | | 0.44 | 1.3 (0.56, 2.0) | | | **<0.001** |
| Abuse/domestic violence | 1.4 (-0.39, 3.2) | 0.12 | 1.8 (-1.1, 4.7) | | | 0.23 | 3.4 (1.1, 5.6) | | | **0.0034** |
| Social support |  |  |  |  |  |  |  |  |  |  |
| number of persons^6^ | 0.70 (-0.16, 1.6) | 0.11 | 0.58 (-0.82, 2.0) | | | 0.41 | 0.85 (-0.24, 1.9) | | | 0.12 |
| satisfaction rating | 0.0071 (-0.046, 0.061) | 0.79 | -0.035 (-0.12, 0.052) | | | 0.43 | -0.027 (-0.094, 0.040) | | | 0.43 |
| F-statistic (*p*-value) | (21, 161) = 4.7 (*p*< 0.001) | | (21, 161) = 2.3 (*p*=0.0023) | | | | (21, 161) = 1.9 (*p*=0.012) | | | |
| Adjusted R^2^ | 0.30 | | 0.13 | | | | 0.097 | | | |

^1^ fear of giving birth; ^2^ worries about bearing a physically or mentally handicapped child; ^3^ concern about own appearance; ^4^ confidence interval; ^5^ body mass index; ^6^ log-transformed (i.e., b represents the change in the PRAQ-R2 score associated with a 1-unit increase in the natural log-transformed variable). Significant *p*-values (*p* < 0.05) are shown in **bold**.
